# A Longitudinal Study of Mental Wellbeing in Students in Aotearoa New Zealand Who Transitioned Into PhD Study

**DOI:** 10.3389/fpsyg.2021.659163

**Published:** 2021-05-20

**Authors:** Taylor Winter, Benjamin C. Riordan, John A. Hunter, Karen Tustin, Megan Gollop, Nicola Taylor, Jesse Kokaua, Richie Poulton, Damian Scarf

**Affiliations:** ^1^Department of Psychology, Victoria University of Wellington, Wellington, New Zealand; ^2^Discipline of Addiction Medicine, Central Clinical School, Faculty of Medicine and Health, University of Sydney, Sydney, NSW, Australia; ^3^Department of Psychology, University of Otago, Dunedin, New Zealand; ^4^National Centre for Lifecourse Research, University of Otago, Dunedin, New Zealand; ^5^Children’s Issues Centre, University of Otago, Dunedin, New Zealand; ^6^Pacific Development Office, University of Otago, Dunedin, New Zealand; ^7^Dunedin Multidisciplinary Health and Development Research Unit, University of Otago, Dunedin, New Zealand; ^8^Office of Student Success, University of Otago, Dunedin, New Zealand

**Keywords:** graduate student, mental health, wellbeing, Warwick-Edinburgh Mental Well-being Scale, longitudinal

## Abstract

Journal editorials, career features, and the popular press commonly talk of a graduate student mental health crisis. To date, studies on graduate student mental health have employed cross-sectional designs, limiting any causal conclusions regarding the relationship between entry into graduate study and mental health. Here, we draw on data from a longitudinal study of undergraduate students in Aotearoa New Zealand, allowing us to compare participants who did, and did not, transition into PhD study following the completion of their undergraduate degree. Using multilevel Bayesian regression, we identified a difference in mental wellbeing between those who entered PhD study and those who did not. This difference, however, was largely due to those not entering PhD study displaying an increase in mental wellbeing. Participants that entered PhD study displayed a small decrease in mental wellbeing, with the posterior distribution of the simple effect heavily overlapping zero. This latter finding was orders of magnitude smaller than one might expect based on previous cross-sectional research and provides an important message; that a marked drop in mental health is not an inevitable consequence of entering graduate study.

## Introduction

There is a plethora of editorials on graduate student mental health in high-impact journals, many with alarming titles such as “Caught in a trap” (Sohn, 2016), “Paying graduate school’s mental toll” (Arnold, 2014), and “The tortuous truth” (Woolston, 2019). The results from Nature’s 2019 survey on student mental health do little to quell the fears of future PhD students. Of the 6,300 early-career researchers surveyed across North America, South America, Africa, Asia, Australia, and Europe, 36% had sought help for anxiety or depression (Woolston, 2019).

In addition to editorials, empirical studies have echoed the alarm (Evans et al., 2018). For example, a survey of 2,279 graduate students, 92% of whom were based at a US institution, identified high prevalence rates of both anxiety and depression. Specifically, 41% reported moderate to severe anxiety and 32% reported moderate to severe depression. After comparing these rates to a general population sample, the authors concluded that there is a mental health crisis in the graduate student population (Evans et al., 2018). One could argue this claim may also hold for undergraduate students, given undergraduate students in the US report higher rates of poor mental health than graduate students (Wyatt and Oswalt, 2013).

Rather than being unique to US institutions, research from Belgium also provides a bleak picture for future PhD students. A survey of 3,659 PhD students revealed that a high proportion of students (32%) reported at least four symptoms of psychological distress (Levecque et al., 2017). For comparison purposes, the PhD student data were compared to a sample of highly educated (Bachelor’s Degree or higher) individuals from the general population. Although lower than the relative prevalence rate reported for students based at US institutions, PhD students had a prevalence of having or developing a psychiatric disorder that was 2.43 times higher than the comparison sample (Levecque et al., 2017). Similar findings have also been reported in French PhD students (Marais et al., 2018).

Although studies attempting to compare prevalence between graduate students and the general population are rare, a number of studies have investigated factors that may predict, or protect against, mental health problems during graduate study (Tong and Song, 2004; McKinzie et al., 2006; Hyun et al., 2007; Kovach Clark et al., 2009; Reilly and Fitzpatrick, 2009; Stubb et al., 2011; Wyatt and Oswalt, 2013; Soysa and Wilcomb, 2015; Huang and Mussap, 2018; Bork and Mondisa, 2019; Byrom et al., 2020). Not surprisingly, these studies highlight the importance of having a good relationship with one’s supervisor (Hyun et al., 2007; Kovach Clark et al., 2009; Byrom et al., 2020), being in a stable financial situation (Tong and Song, 2004; Hyun et al., 2007), and having confidence and clarity regarding one’s future career (Kovach Clark et al., 2009; Byrom et al., 2020). With respect to psychological variables, multiple dimensions of self-efficacy (Tong and Song, 2004; Bork and Mondisa, 2019) and a sense of belonging (Reilly and Fitzpatrick, 2009; Stubb et al., 2011; Arahanga-Doyle et al., 2019; Bork and Mondisa, 2019; Jang et al., 2019) are clear protective factors.

To date, quantitative studies on graduate student mental health have been cross-sectional, limiting any discussion regarding the temporal link between entry into graduate education and mental health. To address this limitation, we used data from the Graduate Longitudinal Study New Zealand (GLSNZ). The GLSNZ conducted baseline sampling of university graduates across all eight New Zealand universities between July and December 2011 (Tustin et al., 2012) and followed up this sample in 2014 (Tustin et al., 2016). In the current study, we compare the mental wellbeing of participants who did, and did not, transition into PhD study following the completion of their undergraduate degree.

## Materials and Methods

For the current study, we focused on the 269 participants who transitioned into PhD study between the baseline survey (2011) and the follow-up survey (2014). All participants completed the Warwick-Edinburgh Mental Well-being Scale (WEMWBS; Tennant et al., 2007), basic demographic information, and a measure of economic strain. To determine whether any changes observed in PhD students were the direct result of the transition into PhD study, we compared PhD students with the 4,230 participants who also graduated in 2011 but did not transition into further study.

### Participants

We collected data from 8,719 participants. We then removed participants who were still enrolled in 2014 but not in PhD study, participants who were in PhD study at baseline, and participants that did not complete all questions used in the analysis, resulting in a final sample of 269 participants who transitioned into a PhD study in 2014 and 4,230 participants who did not enter some form of study in 2014. The PhD entrants had a mean age of 26.7 years old [standard deviation (SD) = 8.4] and were 58% female. Non-entrants had a mean age of 28.6 years old (SD = 10.0) and were 64% female. The study was approved by the University of Otago Human Ethics Committee. Participants provided informed consent at the start of each survey.

### Measures

#### Demographic Information

Participants provided demographic information at baseline, including age, sex, and ethnicity. Additionally, participants were separated into two groups: those who transitioned into PhD study between the 2011 and 2014 surveys (i.e., PhD entrants) and those who did not enter into PhD study after completing their qualification in 2011 (i.e., non-entrants).

#### Economic Strain

The current financial situation of participants was collected using five questions (Tustin et al., 2012, 2016). Questions asked whether participants have trouble meeting financial commitments (e.g., “I have enough money to afford the accommodation I need”). Responses were made on a 1 (strongly disagree) to 5 (strongly agree) scale. We summed responses for all the five items, with a minimum score of five and maximum of 25, with higher scores reflecting less economic strain (Time 1 Cronbach’s *α* = 0.87; Time 2 Cronbach’s *α* = 0.89). The sample used in the analysis had a mean of 18 (SD = 4.5).

#### Mental Wellbeing

We estimated mental wellbeing using the WEMWBS, which consists of 14 items asking about how participants have felt over the last 2 weeks (e.g., “I’ve been able to make up my own mind about things”; Tennant et al., 2007). Responses were made on a 1 (none of the time) to 5 (all of the time) scale. We summed responses for all 14 items, with a minimum score of 14 and a maximum of 70, with higher scores reflecting better mental wellbeing (Time 1 Cronbach’s *α* = 0.91; Time 2 Cronbach’s *α* = 0.92). The sample used in the analysis had a mean of 50 (SD = 7.8).

#### Analysis

**Figure 1:**
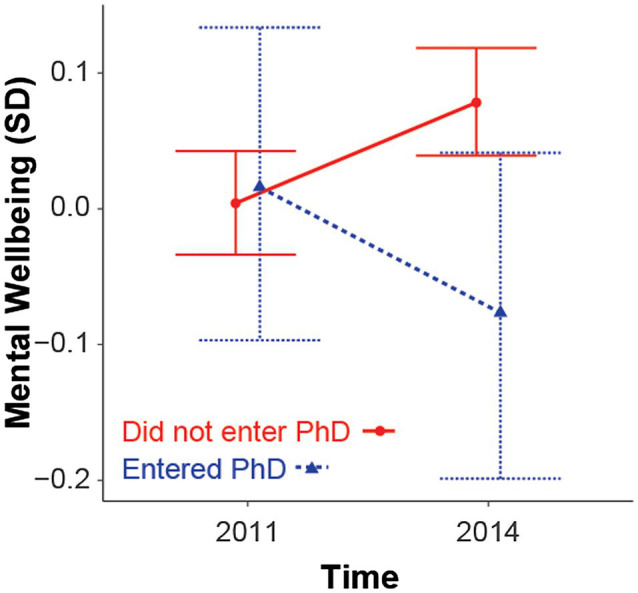
Marginal effects on standardised and centred mental wellbeing in 2011 and 2014 for those who did and did not transitioned into PhD study. Error bars are 95% credible intervals.

All continuous variables were standardized and centered on zero. We then analyzed the data using multilevel Bayesian regression with the brms package in R (Bürkner, 2017). We modeled mental wellbeing as a dependent variable with age, sex, economic strain, time, and PhD entrance as covariates. To test our hypothesis that wellbeing decreases for PhD entrants relative to PhD non-entrants, we included an interaction between time and PhD entry. The interaction term will indicate whether the wellbeing of those who transition into PhD study changes differently over time relative to those who do not transition into PhD study. We then used random intercepts for participants to account for repeated measures. Simple effects were conducted using the emmeans package in R, which has been adapted by the package author for Bayesian analyses but does not return posterior probabilities, as is often the case in Bayesian analyses (Lenth, 2019). The purpose of simple effects was to substantiate our main regression model by reporting at which time point we find a difference between groups (as can be visually interpreted in Figure 1).

#### Construction of Priors

Our priors were based on three previous studies that suggested PhD students had 1.5 standard deviations lower mental wellbeing (Marais et al., 2018), PhD students were six times more likely to experience anxiety or depression (Evans et al., 2018), and PhD students were 2.43 times more likely to develop a psychological condition (Levecque et al., 2017). Taken together and considering some chance that effects are smaller in our Aotearoa New Zealand sample, we used an informed prior for the interaction between time and PhD entrance that took the form of a normal distribution with a standardized mean of −1 and a standard deviation of 0.4. All other variables had a weakly informative normal prior with a mean of zero and a standard deviation of 0.5. In both cases, we intentionally adopted a standard deviation that would give a reasonable chance of an effect being approximately zero (i.e., null). Weakly informative priors were implemented purely for the purposes of minor regularization and to aide in sampling efficiency.

## Results and Discussion

Those who did not transition into a PhD had a mean wellbeing of 49.9 (SD = 7.8), very similar to those who transitioned to PhD who had a mean of 49.7 (SD = 7.6). Similarly, the economic strain was quite similar between the two groups with mean strain in PhD non-entrants of 18.0 (SD = 4.6) compared to PhD entrants with a mean of 17.7 (SD = 4.4).

Multilevel Bayesian regression yielded over a 99.9% posterior probability of an interaction between time and PhD entry (Table 1). Simple effects between the two time points for each group indicated that non-entrants experienced an increase in their level of mental wellbeing in 2014 relative to their level of mental wellbeing in 2011 (time_2014_ − time_2011_ = 0.07, 95% HDI [0.04, 0.10]), whereas PhD entrants presented with lower levels of mental wellbeing in 2014 relative to their levels of mental wellbeing in 2011, but notably, the posterior distribution of the simple effect still heavily overlaps zero (time_2014_ − time_2011_ = −0.1, 95% HDI [−0.21, 0.15]). Thus, in 2011, there was no difference between the two groups (PhD Entrant_TRUE_ − PhD Entrant_FALSE_ = 0.00, 95% HDI [−0.11, 0.12]) but, in 2014, we saw that PhD entrants had lower mental wellbeing than did those who did not transition to PhD study (Figure 1; PhD Entrant_TRUE_ − PhD Entrant_FALSE_ = −0.17, 95% HDI [−0.28, −0.05]). This is a relatively small effect and, at least in part, due to an increase in wellbeing of those who did not enter a PhD study rather than solely due to a decrease in the mental wellbeing of PhD entrants.

**Table 1:** Fixed effects from multilevel Bayesian regression with 95% credible intervals.

	95% credible interval			
	Estimate	Lower	Upper	PP
Intercept	0.01	−0.05	0.03	67.5%
Time (2014)	0.07	0.04	0.10	100.0%
PhD entrant	0.01	−0.11	0.12	53.6%
Sex (female)	−0.04	−0.09	−0.02	96.5%
Economic strain	0.26	0.24	0.28	100.0%
Age	0.10	0.08	0.13	100.0%
Time (2014) × PhD entrant	−0.17	−0.29	−0.05	100.0%

Posterior probability (PP) captures the probability of an effect deviating from zero in its given direction. The analysis uses an informed prior for the time by PhD entrant interaction and a weakly informed prior for all other variables.

The current study represents the first attempt to track mental wellbeing of students as they transition from completing their undergraduate degree into PhD study. PhD entrants displayed a decrease in mental wellbeing between 2011 and 2014. The decrease, however, was extremely small and orders of magnitude smaller than one might expect based on the previous empirical work (Garcia-Williams et al., 2014; Levecque et al., 2017; Evans et al., 2018) and coverage in major journals (Arnold, 2014; Sohn, 2016; Woolston, 2017, 2019). Moreover, there was no evidence that PhD entrants displayed lower levels of mental wellbeing at the baseline assessment, providing no evidence that those who enter a PhD are a self-selecting sample of people with low mental wellbeing.

An important question is why our findings differ from the previous cross-sectional studies on PhD student mental health? One source of difference is the structure of New Zealand PhD programs. Relative to North American programs, New Zealand programs have little (if any) course work. Although they are more similar to the European programs, there are still a number of differences. For example, median time-to-degree in New Zealand is shorter (~4 vs. ~5 years; Spronken-Smith et al., 2018) and completion rates are higher (80 vs. 66%; Spronken-Smith et al., 2018; Hasgall et al., 2019). It is possible, however, that these differences are the product, rather than cause, of better mental health.

A second source of difference is that the current study employed a measure of mental wellbeing (i.e., the WEMWBS), while previous studies have tended to employ measures of depression, anxiety, or general psychological distress. For example, Levecque et al. (2017) employed the GHQ-12. However, although different on the surface, Böhnke and Croudace (2016) recently demonstrated that the GHQ-12 and WEMWBS share a single dimension, with the lack of differentiation suggesting that they provide comparable measures of general health and mental wellbeing. Perhaps, one benefit of the WEMWBS over these other measures is that it takes a more positive approach to assess mental health. Indeed, emerging adults are already viewed through a deficit lens (Trzesniewski and Donnellan, 2014), and reports on the prevalence of depression, anxiety, and general psychological distress of PhD students may contribute to this general depiction. At the very least, studies that assess mental health in PhD students should complement their analysis with a strength-based or mental wellbeing approach.

## Conclusion

Rather than representing limitations, the above differences should be thoroughly investigated to determine whether they are a consequence, or a cause, of good mental wellbeing. If program differences prove to be causative, they may be utilized as a pathway to improve the mental health of graduate students in other countries. Above all, the current study demonstrates that a marked drop in mental wellbeing is not an inevitable consequence of entry into graduate study.

## Data Availability Statement

The data analyzed in this study are subject to the following licenses/restrictions: Researchers can request the data and code used in the analyses by contacting enquiries@glsnz.org.nz(https://www.glsnz.org.nz/). Requests to access these datasets should be directed to https://www.glsnz.org.nz/.

## Ethics Statement

The studies involving human participants were reviewed and approved by the University of Otago Human Ethics Committee. The patients/participants provided their written informed consent to participate in this study.

## Author Contributions

KT, MG, NT, JK, and RP designed and performed the research. TW, BR, and DS analyzed the data. KT, TW, BR, and DS wrote the paper. All authors contributed to the article and approved the submitted version.

### Conflict of Interest

The authors declare that the research was conducted in the absence of any commercial or financial relationships that could be construed as a potential conflict of interest.
